# Supplementary material for: Syngap1 regulates the synaptic drive and membrane excitability of Parvalbumin-positive interneurons in mouse auditory cortex
Source: eLife. 2025 Aug 14;13:RP97100. doi: 10.7554/eLife.97100 (PMC12352866; doi:10.7554/eLife.97100)
Supplement: Supplementary file 1. [file elife-97100-supp1.doc]

**Supplementary File 1.** Membrane properties of BC-broad Vs BC-short in control mice.

|  | **BC-broad**  **(n=21)**  **12 animals** | **BC-short**  **(n=12)**  **9 animals** | **LMM** |
| --- | --- | --- | --- |
| **Resting membrane potential (mV)** | -69.47 ± 0.87 | -72.33 ± 1.69 | F=2.511  p=0.126 |
| **Input resistance (MΩ )** | 297.14 ± 19.98 | 237.66 ± 19.98 | F=4.830  p=0.036* |
| **Membrane capacitance (pF)** | 26.66 ± 1.30 | 33.08 ± 1.09 | F=10.973  p=0.02** |
| **Membrane time constant (ms)** | 7.95 ± 0.67 | 7.75 ± 0.58 | F=0.042  p=0.838 |
| **Rheobase current (pA)** | 123.80 ± 7.76 | 138.33 ± 12.42 | F=1.020  p=0.323 |
| **AP threshold (mV)** | -42.11 ± 1.31 | -43.38 ± 1.63 | F=0.373  p=0.546 |
| **AP amplitude (mV)** | 62.42 ± 1.85 | 61.43 ± 1.94 | F=0.040  p=0.843 |
| **AP latency (ms)** | 28.63 ± 4.63 | 36.36 ± 7.91 | F=0.871  p=0.358 |
| **fAHP (mV)** | -15.19 ± 0.67 | -16.05 ± 1.06 | F=0.551  p=0.464 |
| **I_h_ sag (mV)** | 0.77 ± 0.07 | 0.72 ± 0.12 | F=0.093  p=0.763 |
| **SAAR** | 0.75 ± 0.01 | 0.81 ± 0.02 | F=3.644  p=0.066 |
| **FFAR** | 1.38 ± 0.03 | 1.31 ± 0.06 | F=0.904  p=0.353 |
| **Firing frequency at 2x rheobase current (Hz)** | 75.52 ± 5.74 (n=20) | 100.83 ± 7.55 | F=7.082  p=0.012* |
| **Fmax (Hz)** | 144.58 ± 6.73 (n=16) | 191.97 ± 10.48 (n=11) | F=16.315  p<0.001*** |
| **Fss (Hz)** | 87.27 ± 5.04 (n=16) | 124.23 ± 9.32 (n=11) | F=15.344  p<0.001*** |
