# Supplementary material for: Syngap1 regulates the synaptic drive and membrane excitability of Parvalbumin-positive interneurons in mouse auditory cortex
Source: eLife. 2025 Aug 14;13:RP97100. doi: 10.7554/eLife.97100 (PMC12352866; doi:10.7554/eLife.97100)
Supplement: Supplementary file 2. [file elife-97100-supp2.doc]

**Supplementary File 2. sEPSCs in SST+ cells from control Vs SST+ cells from cHet mice.**

|  | **Control**  **(n=17, 10 anim.)** | **cHet**  **(n=10, 8 anim.)** | **LMM** |
| --- | --- | --- | --- |
| **sEPSC ampl.** | 17.63 ± 1.55 pA | 11.63 ± 1.17 pA | F=7.850  p=0.010* |
| **AUC sEPSC** | 90.75 ± 12.23 pA x ms | 53.56 ± 6.05 pA x ms | F=4.942  p=0.047* |
| **sEPSC time interval** | 209.07 ± 52.48 ms | 390.24 ± 115.62 ms | F=2.858  p=0.103 |
| **ΔQ * f** | 0.79 ± 0.19 | 0.26 ± 0.06 | F=4.438  p=0.045* |
| **Rise time sEPSC** | 0.53 ± 0.04 ms | 0.62 ± 0.11 ms | F=0.546  p=0.471 |
| **Decay time**  **sEPSC** | 3.92 ± 0.49 ms | 3.51 ± 0.59 ms | F=0.295  p=0.594 |
